# Allosteric regulation by cooperative conformational changes of actin filaments drives mutually exclusive binding with cofilin and myosin

**DOI:** 10.1038/srep35449

**Published:** 2016-10-20

**Authors:** Kien Xuan Ngo, Nobuhisa Umeki, Saku T. Kijima, Noriyuki Kodera, Hiroaki Ueno, Nozomi Furutani-Umezu, Jun Nakajima, Taro Q. P. Noguchi, Akira Nagasaki, Kiyotaka Tokuraku, Taro Q. P. Uyeda

**Affiliations:** 1 Biomedical Research Institute, National Institute of Advanced Industrial Science and Technology, Tsukuba, Ibaraki 305-8562, Japan; 2 Department of Physics, Faculty of Science and Engineering, Waseda University, Tokyo 169-8555, Japan; 3 Graduate School of Life and Environmental Sciences, University of Tsukuba, Ibaraki 305-8572, Japan; 4 Miyakonojo National College of Technology, Miyakonojo, Miyazaki 885-8567, Japan; 5 Department of Physics and Bio-AFM Frontier Research Center, Kanazawa University, Kanazawa, Ishikawa 920-1192, Japan; 6 PRESTO, Japan Science and Technology Agency, Kawaguchi, Saitama 332-0012, Japan; 7 Department of Applied Sciences, Muroran Institute of Technology, Muroran, Hokkaido 050-8585, Japan

## Abstract

Heavy meromyosin (HMM) of myosin II and cofilin each binds to actin filaments cooperatively and forms clusters along the filaments, but it is unknown whether the two cooperative bindings are correlated and what physiological roles they have. Fluorescence microscopy demonstrated that HMM-GFP and cofilin-mCherry each bound cooperatively to different parts of actin filaments when they were added simultaneously in 0.2 μM ATP, indicating that the two cooperative bindings are mutually exclusive. In 0.1 mM ATP, the motor domain of myosin (S1) strongly inhibited the formation of cofilin clusters along actin filaments. Under this condition, most actin protomers were unoccupied by S1 at any given moment, suggesting that transiently bound S1 alters the structure of actin filaments cooperatively and/or persistently to inhibit cofilin binding. Consistently, cosedimentation experiments using copolymers of actin and actin-S1 fusion protein demonstrated that the fusion protein affects the neighboring actin protomers, reducing their affinity for cofilin. In reciprocal experiments, cofilin-actin fusion protein reduced the affinity of neighboring actin protomers for S1. Thus, allosteric regulation by cooperative conformational changes of actin filaments contributes to mutually exclusive cooperative binding of myosin II and cofilin to actin filaments, and presumably to the differential localization of both proteins in cells.

Actin filaments perform a variety of important functions in eukaryotic cells, ranging from cytokinesis, lamellipodial extension, adhesion, intracellular transport and nuclear functions, in a manner dependent on the interaction with specific actin binding proteins (ABPs). In a number of cases, researchers have successfully explained the regulation of ABPs by local biochemical signaling, such as phosphorylation and changes in the concentration of signaling molecules. However, not all localized activities of ABPs are fully explained by specific biochemical signaling. Thus, the spatial and temporal regulation of the activity of each ABP in a cell remain major unresolved issues in cell biology.

Meanwhile, a number of biochemical and biophysical studies have established that binding of ABPs induces specific conformational changes in actin filaments, which are in certain cases known to propagate along the filament, i.e., cooperative conformation changes. The pioneering work of Oosawa and colleagues demonstrated that the addition of two-headed soluble fragment of skeletal muscle myosin (heavy meromyosin or HMM) increases the fluorescence intensity of labeled actin, and that this effect became saturated in the presence of a 1/20 molar ratio of HMM to actin^1^. Subsequently, a number of studies reported similar, myosin-induced cooperative conformational changes in actin filaments detected using different methods (e.g., refs 2,3). Furthermore, a large body of evidence has accumulated that demonstrates that cofilin binding also changes the structure of actin protomers in a cooperative manner. For instance, cofilin binding super-twists the helix of actin filaments by ~25%, involving changes in the atomic structure of each actin protomer^4^,^5^,^6^,^7^, and this conformational change is propagated to a neighboring bare zone^4^,^8^ on the pointed end side^7^. A single bound cofilin molecule affects the structure of ~100 actin protomers in the filament^9^,^10^,^11^, and increases the affinity for a second cofilin molecule within the ~65 nm vicinity^12^. Furthermore, binding of certain ABPs to actin filaments is cooperative. Cofilin forms tight clusters along the filament, leaving other parts of the filament mostly bare^4^,^5^,^7^,^13^. HMM also binds to actin filaments cooperatively under certain conditions^14^,^15^. In the presence of low concentrations of ATP, HMM is sparsely bound along the entire length of a fraction of actin filaments, while leaving other filaments bare^15^. In both cases, HMM and cofilin molecules in clusters along actin filaments do not directly contact with each other^6^,^15^. Thus, the cooperative binding of HMM and that of cofilin to actin filaments most likely involve cooperative conformational changes of actin protomers within the filaments.

Stretching actin filaments untwists actin filaments^16^,^17^. This mechanosensitivity of actin filaments might provide an additional mechanism to regulate ABP binding^18^,^19^. For instance, stretched actin filaments are resistant to the severing activity of cofilin both *in vivo* and *in vitro*^20^, whereas an *in vivo* study suggested that stretching actin filaments increases the affinity for myosin II^19^.

This raises an interesting possibility that cooperative conformational changes of actin filaments may contribute to the determination of the function of actin filaments by recruiting specific ABPs^15^,^18^,^19^,^21^,^22^. Furthermore, if two ABPs induce different cooperative structural changes in actin filaments, cooperative binding of those two ABPs should be mutually exclusive, leading to functional differentiation of the filaments. Here, we tested this latter prediction experimentally using two different approaches. Myosin II and cofilin were chosen as the candidates of mutually incompatible ABPs, not only because they are major ABPs, but also because these two proteins cause opposite changes in the helical twist of actin filaments, show opposite responses to the stretching of actin filaments, and have different intracellular localizations.

## Results

### Fluorescence microscopic examination in the presence of low concentrations of ATP

We previously found that when *Dictyostelium discoideum (Dd*) HMM was fused with GFP (HMM-GFP), and was mixed with excess rabbit skeletal (sk) actin filaments labeled with 1/20 (mol/mol) rhodamine phalloidin in the presence of 0.1~1 μM ATP, certain filaments were diffusely labeled with GFP along the entire lengths, whereas other filaments were left unbound^15^. This was termed weak cooperativity in the sense that several unbound actin protomers were present between bound HMM molecules. Use of low concentration of ATP was essential to obtain the weak cooperative binding of HMM to actin filaments; in the absence of ATP, HMM-GFP was uniformly bound along all actin filaments because the affinity was too high, and in the presence of higher concentration of ATP, no binding was detected during the observation period. We speculated that the low concentration of ATP allows dissociation-association cycles to facilitate relocation of HMM-GFP to sections of actin filaments with a high affinity for HMM, and eventually stabilizes the binding when depleted by the ATPase activity^15^. In a similar experiment, Suarez *et al*. reported that when fluorescently labeled yeast cofilin was mixed with actin filaments, it formed tight clusters along the filaments while apparently leaving other sections of the filaments bare^13^.

**Figure 1:**
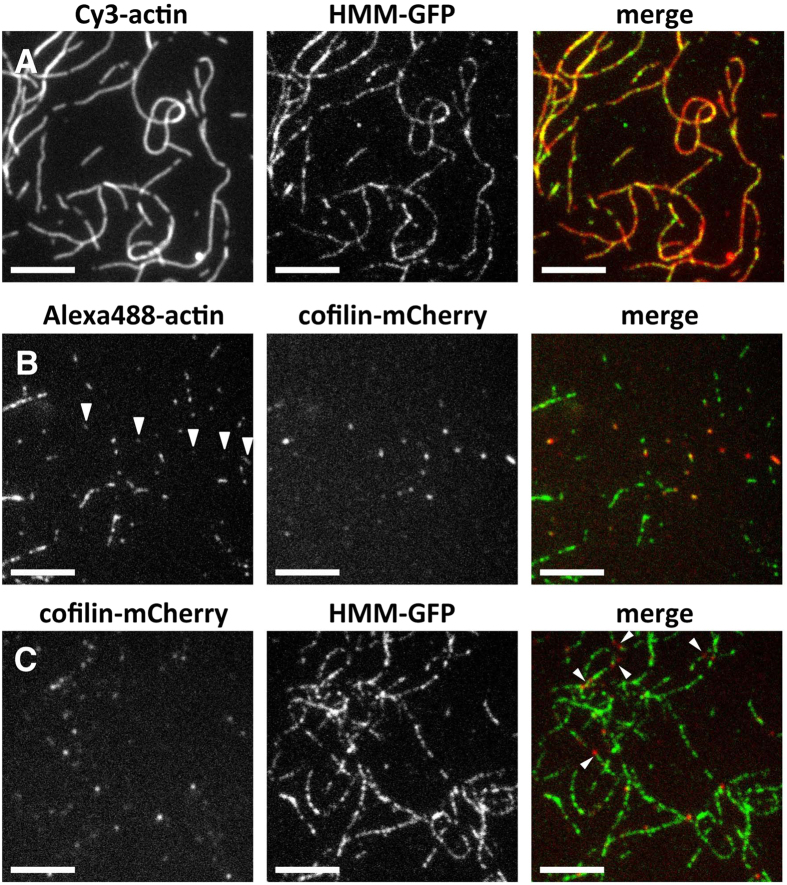
Fluorescence microscopic observation of binding of *Dd* HMM-GFP and *Hs* cofilin-mCherry to *Dd* actin filaments. In all experiments, actin filaments were loosely immobilized on a positively-charged lipid bilayer and the binding reaction was performed in F-buffer 1 containing 0.2 μM ATP. (**A**) Cooperative binding of 7 nM HMM-GFP along Cy3-labeled actin filaments, forming short clusters or puncta along the actin filaments. (**B**) Cooperative binding of 80 nM cofilin-mCherry to Alexa488-labeled actin filaments. Cofilin-mCherry formed tight clusters along filaments. Alexa488 fluorescence of filaments bound with cofilin-mCherry was weaker (arrowheads), presumably due to quenching by FRET with mCherry. (**C**) Simultaneous addition of 7 nM HMM-GFP and 80 nM cofilin-mCherry to unlabeled actin filaments. Because the concentrations of actin filaments and HMM-GFP were identical to those in (**A**), most of the filaments were labeled by HMM-GFP in a punctate manner. Other shorter filaments were labeled only by cofilin-mCherry. Additionally, some filaments were labeled by both fluorescent proteins in an alternating punctate manner (arrowheads). Scale bars: 5 μm.

We thus queried what would happen if fluorescent-cofilin and HMM-GFP coexisted in actin solutions in the presence of low concentrations of ATP. We first repeated our previous experiments of weak cooperative binding of HMM-GFP to excess actin filaments, except that in the present study we used *Dd* actin filaments that were loosely immobilized on a positively charged surface in advance. Furthermore, because phalloidin interferes with cofilin binding^23^, actin was visualized by chemical labeling with Cy3-succinimide and used without stabilization with phalloidin. The pH of the solution was reduced from 7.4 to 6.5, because slightly acidic condition suppresses the severing activity of cofilin^23^,^24^,^25^ and allows the examination of cofilin binding in the same buffer. Despite these changes in experimental conditions, 7 nM *Dd* HMM-GFP was bound along limited sections of the actin filaments while apparently leaving other sections of the filament bare (Fig. 1A). We noticed that, however, the binding of HMM-GFP along the filaments was more punctate than in our earlier observation^15^. We suspect that this difference was primarily due to the use of *Dd* actin instead of sk actin.

We next added 80 nM human (*Hs*) cofilin fused with mCherry to *Dd* actin filaments labeled by Alexa488-succinimide and loosely adsorbed on a positively charged surface. Consistent with an earlier report^13^, sections of filaments were bound with *Hs* cofilin-mCherry, while other sections were apparently bare (Fig. 1B). Filaments were shorter than those incubated with HMM-GFP, due to residual severing activity of cofilin.

Finally, a mixture of 80 nM *Hs* cofilin-mCherry and 7 nM *Dd* HMM-GFP was allowed to interact with *Dd* actin filaments loosely adsorbed on a positively charged surface (Fig. 1C). Under this experimental condition, the majority of filaments were labeled by HMM-GFP in a punctate manner, and cofilin-mCherry formed separate puncta. Importantly, punctate staining by HMM-GFP and cofilin-mCherry never overlapped. These results suggest that cooperative bindings of cofilin and HMM to actin filaments are mutually exclusive.

### TIRFM observation of the inhibitory effect of S1 on cofilin binding in the presence of 0.1 mM ATP

**Figure 2:**
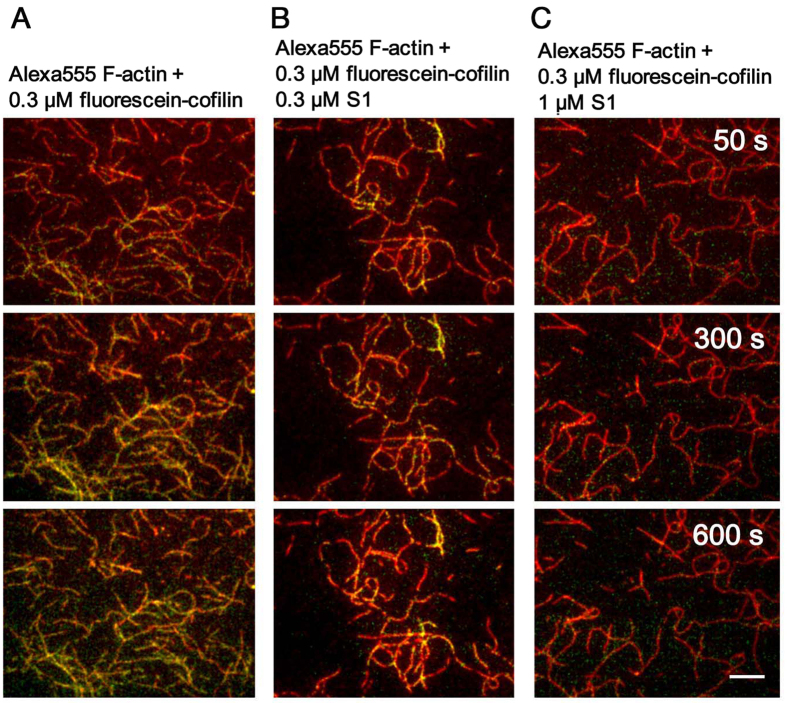
TIRF microscopic observation of inhibitory effects of sk S1 on binding of chicken cofilin to sk actin filament in the presence of 0.1 mM ATP. Merged TIRF images of Alexa555-actin filaments (red) and fluorescein-cofilin (green) of the same field, captured at different indicated times, shown in s, after the addition of 0.3 μM fluorescein-cofilin without (**A**) or with 0.3 μM (**B**) or 1 μM S1 (**C**). Bar: 5 μm.

**Figure 3:**
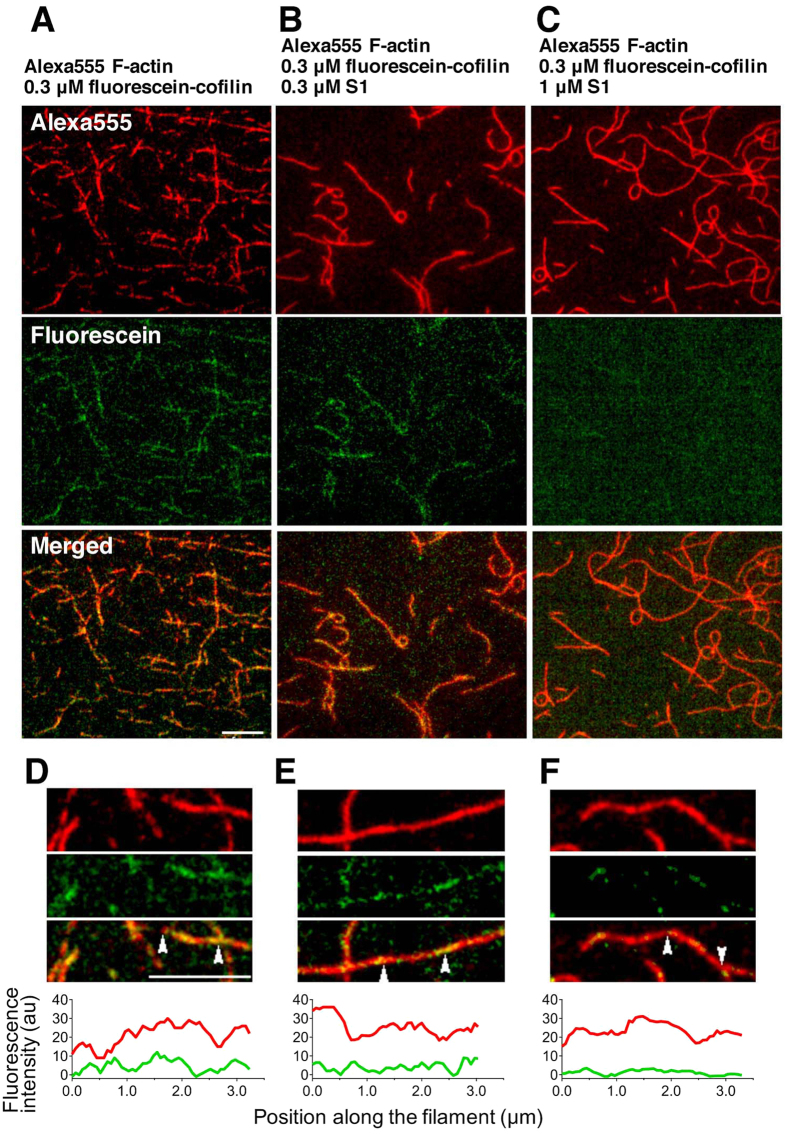
TIRF microscopic observation of inhibitory effects of sk S1 on binding of chicken cofilin to sk actin filament in presence of 0.1 mM ATP. (**A–C**) are fluorescence images of Alexa555-sk actin filaments and fluorescein-cofilin and the merge image, captured 10 min after the addition of 0.3 μM fluorescein-cofilin without (**A**) or with 0.3 μM (**B**) or 1 μM S1 (**C**). Bar: 5 μm. (**D–F**) are enlarged views of a different field of the same sample as (**A–C**), respectively. The top and middle rows show Alexa555 and fluorescein images, and the bottom row shows the merged image, respectively. Bar: 5 μm. The graphs at the bottom show fluorescence intensity profiles of Alexa555 (red) and fluorescein (green) after background subtraction along the filament segments flanked by the two arrowheads in the merged images immediately above.

The mutually exclusive binding of cofilin and HMM to actin filaments could be due either to direct competition of binding sites on actin protomers or to allosteric cooperative conformational changes of actin filaments. To distinguish these two possibilities, binding of cofilin to actin filaments was observed in the presence of the motor domain or subfragment 1 (S1) of sk myosin II and ATP by total internal reflection fluorescence microscopy (TIRFM). The concentration of ATP in this experiment was 0.1 mM, 500-fold higher than that used in the experiment shown above, so that a saturating concentration of ATP was maintained during the observation. Two extra Cys residues at the N-terminus of chicken cofilin 2 were labeled with iodoacetamide fluorescein, and the previous biochemical characterization confirmed that the resultant fluorescein-cofilin binds to actin filaments normally and severs them in a pH-dependent manner^26^. Sk actin filaments were labeled with Alexa555-succinimide, and were purified by a cycle of depolymerization-polymerization. Cosedimentation experiment confirmed that fluorescein-cofilin interacts normally with Alexa555-actin filaments (Supplementary Fig. S1). Alexa555-actin filaments were loosely immobilized on positively charged inner surfaces of flow cells. Following introduction of 0.3 μM fluorescein-cofilin in 0.1 mM ATP, 40 mM KCl and 20 mM Pipes pH 6.8, the fluorescence of Alexa555 and fluorescein were observed by TIRFM. Spots of fluorescein were visible along actin filaments at 50 s after the addition of fluorescein-cofilin, and the number and size of the fluorescein spots increased over the time course of 10 min (Figs 2A and 3A). In contrast, very few fluorescein-cofilin clusters formed, even after 10 min when 1 μM S1 was added 5 min prior to the addition of fluorescein-cofilin (Figs 2C and 3C). A weaker inhibitory effect on cofilin cluster formation was observed in the presence of 0.3 μM S1 (Figs 2B and 3B). A semi-quantitative evaluation of the inhibitory effects by S1 is summarized in Fig. 3D–F and Supplementary Table S1. Under the present condition (0.1 mM ATP, 40 mM KCl, pH 6.8), binding of S1 to actin filaments is transient, and the majority of actin protomers in the filaments should be free of bound S1 at any given moment, as confirmed by live imaging by high speed atomic force microscopy (Supplementary Fig. S2 and Supplementary Videos S1 and S2). It is therefore obvious that inhibition of cofilin cluster formation by S1 in the presence of ATP does not depend on direct competition of binding sites on actin protomers, leading us to conclude that the inhibition of cofilin cluster formation is primarily allosteric and depends on cooperative conformational changes of actin filaments.

### Cosedimentation assays using fusion proteins

The mechanism of mutually exclusive cooperative binding of cofilin and HMM to actin filaments was further explored by cosedimentation assays. Initially, we carried out a series of standard cosedimentation experiments using sk actin filaments, sk S1 and human cofilin. Unexpectedly, however, we were unable to find a condition under which S1 in the presence of ATP significantly reduced the amount of cofilin co-pelleting with actin filaments (e.g., Supplementary Fig. S3). One major difference between these cosedimentation experiments and the TIRFM (Fig. 2) and AFM (Supplementary Fig. S2) experiments is the molar ratio between S1 and actin in the reaction mixtures. In the two microscopic experiments, concentrations of actin were very low, in the order of 10 nM, such that concentration of S1, 0.3~1 μM, was 30~100-fold higher than that of actin, whereas in the cosedimentation experiments, the concentrations of S1 were less than that of actin. We speculated that this may be the reason why S1 in the presence of ATP inhibited cofilin binding in the microscopic experiments but not in the cosedimentation experiments, considering the kinetics of the actin-activated ATPase cycle of S1 (Supplementary Fig. S4). If so, this potential problem can be circumvented by using S1 that is physically tethered to actin protomers, as tethering would increase the effective concentration of S1 around actin filaments.

**Figure 4:**
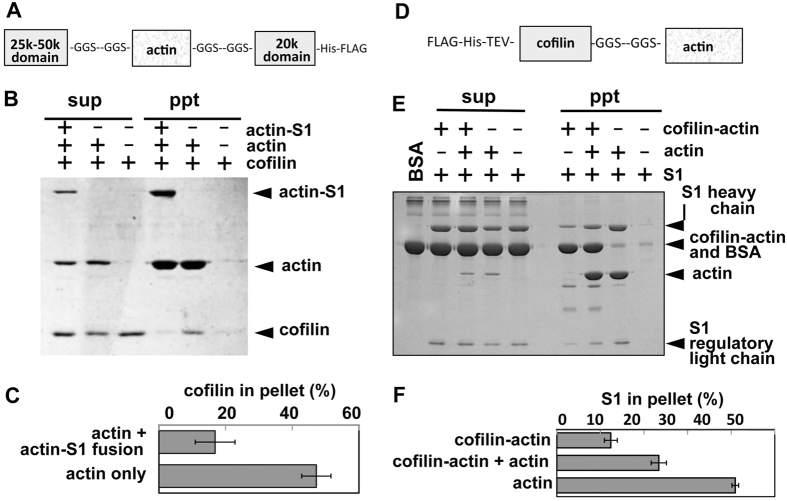
Cosedimentation experiments to assay the affinity of cofilin for copolymer of actin and actin-S1 fusion protein (**A–C**), and that of S1 for copolymer of actin and cofilin-actin fusion protein (**D–F**). (**A**) Schematic structure of actin-S1 fusion protein. (**B**) Cosedimentation of 3 μM *Dd* cofilin with 3 μM *Dd* actin homopolymer or copolymer (3 μM *Dd* actin and 1.5 μM actin-S1) in the presence of 70 mM KCl and 2 mM ATP at pH 6.5. Supernatant (sup) and pellet (ppt) fractions after ultracentrifugation were analyzed by SDS-PAGE. (**D**) Schematic structure of cofilin-actin fusion protein. (**E**) Cosedimentation of 1.5 μM *Dd* S1 with 5 μM cofilin-actin fusion protein homopolymer, copolymer (5 μM cofilin-actin, 4 μM *Dd* actin) or 4 μM *Dd*, actin homopolymer in the presence of 50 mM KCl, 1 mM ADP and 0.2 mM ATP at pH 6.5. Supernatant (sup) and pellet (ppt) fractions after ultracentrifugation were analyzed by SDS-PAGE. Two-fold larger amounts of pellet fractions were loaded. (**C,F**) Densitometric quantitation showing mean ± SD from three independent experiments.

Actin-S1 fusion protein has the whole *Dd* actin polypeptide inserted in loop 2 of S1 of *Dd* myosin II using two Gly-based 18 amino acid-residue linkers (Fig. 4A)^27^. Loop 2 is an actin binding site of S1, and this fusion was intended to mimic the natural actin-S1 complex. Our previous characterization^27^ showed that the actin-S1 fusion protein was unable to polymerize on its own, which was contrary to the initial expectation. Nonetheless, it was able to copolymerize with sk actin, and the copolymerization significantly enhanced the MgATPase activity of the S1 moiety, albeit not to the level of V_max_. Furthermore, copolymer of actin-S1 fusion protein with actin was indistinguishable from actin filaments sparsely bound with sk S1 by electron microscopy. We therefore concluded that the actin-S1 fusion protein in copolymer with actin mimics certain aspects of frequently interacting actin and S1 with very high local concentration of S1. Based on those two rationales, we attempted to reproduce inhibition of cofilin binding to actin filaments by cooperative conformational changes induced by S1 in the presence of ATP using the actin-S1 fusion protein in cosedimentation experiments.

The affinity of *Dd* cofilin for copolymers of actin-S1 fusion protein and *Dd* actin was compared with that for homopolymers of *Dd* actin at pH 6.5 and a KCl concentration of 70 mM, the conditions under which cofilin binds actin filaments without rapidly severing them^23^,^24^,^25^. Actin-S1 fusion protein in copolymers strongly inhibited binding of cofilin to neighboring actin protomers (Fig. 4B,C). The molar ratio of actin-S1 fusion protein to actin in this experiment was 1:2, implying that each actin-S1 fusion protein affected more than two neighboring actin protomers.

In the second experiment, we used another fusion protein, in which *Dd* actin was fused at the C-terminus of *Dd* cofilin via a Gly-based 14 amino acid residue linker (Fig. 4D). Similar to the actin-S1 fusion protein, this cofilin-actin fusion protein copolymerizes with *Dd* actin and affects the structure of neighboring actin protomers^8^. Within the copolymer, the fusion protein did not segregate from actin to form tight clusters, although its distribution within copolymer was not necessarily uniform^8^. This is a clear advantage over using actin filaments bound with cofilin because cofilin binds to actin filaments highly cooperatively and forms tight clusters, which would hinder quantitative interpretation of the impact of cofilin binding at a given molar ratio to actin.

The affinity of *Dd* S1 for copolymers of cofilin-actin fusion protein and *Dd* actin was compared with that for homopolymers of *Dd* actin in the presence of 0.2 mM ATP, 1 mM ADP, and 50 mM KCl at pH 6.5. Under this condition, S1 binds actin filaments with an intermediate affinity (Fig. 4E,F). Notably, much less S1 cosedimented with copolymers of cofilin-actin fusion protein and actin than with actin homopolymers.

Actin protomers in copolymers with actin-S1 fusion protein retained their ability to bind *Dd* S1 and skeletal HMM (Supplementary Fig. S5), and actin protomers in copolymers with cofilin-actin retained their ability to bind cofilin cooperatively^8^. These results imply that the inhibition of cofilin or S1 binding by actin-S1 or cofilin-actin fusion protein is specific to a certain class of ABPs, rather than due to general structural disturbance caused by the fusion proteins.

## Discussion

### Mechanism of mutually exclusive cooperative binding of myosin II and cofilin to actin filaments

We have shown that cooperative bindings of cofilin and the motor domain of myosin II to actin filaments are mutually exclusive (Figs 1 and 4). The mutually inhibitory binding of the motor domain of myosin II and cofilin to actin filaments has been reported in a number of studies, and was attributed to direct competition for binding sites on actin^28^,^29^,^30^,^31^,^32^. However, our TIRFM observation clearly indicated that, at least when cofilin cluster formation was inhibited by S1 in the presence of ATP, direct competition of binding sites does not play a major role. This is because in the presence of 0.1 mM ATP, binding of S1 to actin filaments is transient, and the majority of actin protomers in filaments are free of bound S1 at any given moment (ref. 33 and Supplementary Fig. S2 and Supplementary Video S2). Our cosedimentation experiments also showed that the inhibition of cofilin binding to copolymers of actin and actin-S1 fusion protein, and the inhibition of S1 binding to copolymers of actin and cofilin-actin fusion protein do not require direct competition for binding sites on actin protomers. This is because the same concentration of actin protomers was available for binding in both the copolymer and the control actin homopolymer filaments (Fig. 4). Modeling using published coordinates of cofilin-decorated actin filaments and an actin-S1 rigor complex indicated that bound cofilin and bound S1 molecules do not physically interfere with each other’s binding at an adjacent binding site (Supplementary Fig. S6). Rather, this inhibition most likely depends primarily on allosteric and cooperative conformational changes of the actin protomers, which were induced by free cofilin, HMM or S1 in the microscopic binding experiments (Figs 1–3) or by the neighboring fusion protein in the cosedimentation experiments (Fig. 4). Notably, in the case of S1-dependent inhibition of cofilin cluster formation in the presence of ATP, binding of S1 to actin filaments was transient and the majority of actin protomers in filaments were free of bound S1 molecules (Supplementary Fig. S2 and Supplementary Video S2). This suggests that the inhibitory effect of transient binding of S1 propagates over a long distance. Alternatively, each bound S1 molecule may affect fewer neighboring actin protomers, but the inhibitory effect persisted after the S1 molecule dissociated.

Implied in this hypothesis is that the conformational changes of the actin protomers induced by myosin II and cofilin are different. Cofilin binding significantly supertwists the helix of actin filaments^4^,^5^,^6^,^7^. In contrast, the actin helix slightly untwists when myosin motor domain binds in the absence of ATP^34^,^35^. This is consistent with the above prediction, but further analysis is needed to reveal the conformational changes of actin filaments induced by the myosin motor domains in the presence of ATP.

### Physiological implications of mutually exclusive cooperative binding of myosin II and cofilin to actin filaments

In the amoeboid cells of the cellular slime mold *Dictyostelium discoideum*, cofilin is enriched in the lamellipodia^36^ to recycle actin for continuous polymerization at the leading edge, while myosin II is enriched in the posterior cortex^37^,^38^ to drive retraction^39^. Below is a brief summary of the plausible biochemical signaling involved in the localization of cofilin and myosin II in motile cells.

The activity of mammalian cofilin has been shown to be regulated by three independent biochemical mechanisms. First, activity is suppressed by the phosphorylation of Ser3^40^. However, overexpression of constitutively active S3A cofilin did not inhibit cell motility^41^,^42^. Second, cofilin is sequestered to the plasma membrane in an inactive form by phosphatidylinositol 4,5-bisphophate (PIP_2_) and this inhibition is reversed by phospholipase C, which degrades PIP_2_^43^. However, phospholipase C is not required for chemotaxis in *Dictyostelium*^44^. Finally, the severing activity of cofilin is enhanced at a higher pH, which, at the leading edge of motile cells, may be required for local activation of cofilin^40^. Consistent with this view, inhibition of the Na^+^/H^+^ exchanger (NHE1) in neutrophils and *Dictyostelium* amoebae impaired the elevation of intracellular pH and chemotaxis^45^,^46^. However, the motility defect in NHE1-null *Dictyostelium* amoeba was suppressed by the overexpression of Aip1, which binds and increases the activity of cofilin^47^. Thus, there is no compelling evidence to show that Ser3 phosphorylation, inactivation by PIP_2_ binding, or activation by increased pH is singly essential for localized activation of cofilin in *Dictyostelium*.

The interaction between actin filaments and cofilin is influenced by the nucleotide bound to actin protomers. Actin protomers with bound ATP or ADP and phosphate have lower affinity for cofilin than those carrying ADP^48^,^49^, so that the newly polymerized actin filaments are less prone to severing by cofilin *in vitro*. This has been suggested to play a role to protect newly polymerized actin filaments close to the leading edge of lamellipodia^50^. However, this mechanism cannot explain why cortical actin filaments along the sides and in the posterior region do not bind cofilin.

The assembly of *Dictyostelium* myosin II into filaments is suppressed by phosphorylation of the heavy chain^51^,^52^. However, filaments of mutant myosin IIs, from which this phosphorylation regulation was removed, were still localized in the posterior region^52^, eliminating the possibility of essential roles of this phosphorylation regulation in the localization of *Dictyostelium* myosin II.

**Figure 5:**
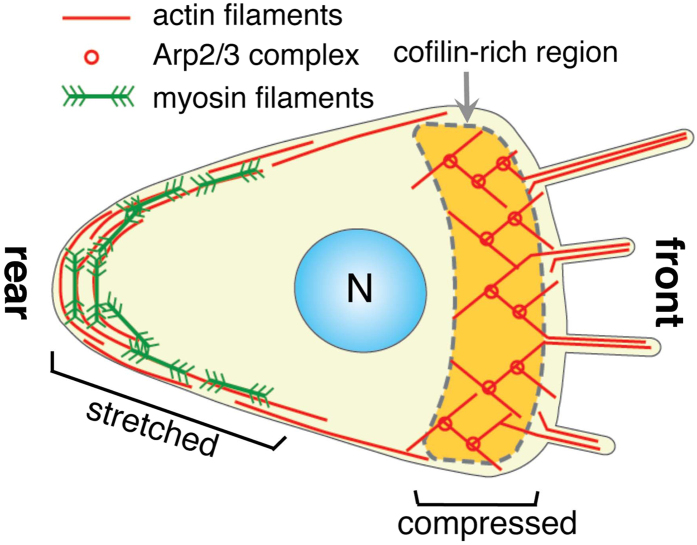
Schematic drawing of actin structures and forces acting on actin filaments in a migrating *Dictyostelium* cell. Note that, unlike in crawling higher animal cells (e.g., ref. 63), myosin II is absent in the anterior region in a migrating *Dictyostelium* cell^38^. In higher animal cells, tropomyosin has been implicated in specifying the localization of ABPs^64^. However, a discernable tropomyosin gene has not been discovered in the fully-sequenced genome of *Dictyostelium*, suggesting that tropomyosin is dispensable to properly localize ABPs differentially in polarized amoeboid cells of simpler organisms.

Thus, despite extensive work, the essence of the molecular mechanism of anterior localization of cofilin and posterior localization of myosin II in migrating *Dictyostelium* cells is still obscure. We propose that different physical conditions of actin filaments combined with the mutually exclusive cooperative binding of cofilin and myosin II to actin filaments contribute to regulating the localization of those ABPs in migrating *Dictyostelium* cells (Fig. 5), as detailed next.

Actin filaments in the posterior cortex of migrating cells interact with myosin II filaments to drive contraction of the rear end, so that those actin filaments are mechanically stretched. An X-ray diffraction study of contracting muscle^17^ and a molecular dynamics simulation^16^ demonstrated that stretched actin filaments have an untwisted and longer helical pitch than control filaments. Independent of force generation, binding of the myosin motor alone changes the structure of actin filaments and untwists the helical pitch^34^,^35^, implying that myosin motors have a higher affinity for untwisted filaments. In the posterior region, therefore, increased tension, an untwisted helical structure, increased binding of myosin II filaments, and the resulting increased tension would form a positive feedback loop, stabilizing the established front-rear cell polarity. Cofilin would be excluded from such established local positive feedback loops, either because cofilin has a lower affinity for stretched actin filaments^20^ and/or because cofilin cannot bind to actin filaments interacting with myosin motor domains (Figs 1C, 2C, 3C,F and 4B). In the present study, inhibition of actin binding of cofilin by S1 in the presence of saturating concentration of ATP was observed only when the molar ratio of S1 to actin was very large (TIRFM and AFM) or when actin-S1 fusion protein was used (cosedimentation). *In vivo*, clustering of motor domains in filaments of myosin II would contribute to increase the local concentration of the myosin motor domain near interacting actin filaments.

Along the leading edge of migrating cells, Arp2/3-dependent polymerization of actin filaments pushes the cell membrane forward^53^, so that those actin filaments experience a compressive force. This compressed state of the filaments would favor cofilin binding^20^. The abundance of cofilin binding and the supertwisted conformation of the filaments would contribute to exclusion of myosin II from cooperatively binding to actin filaments in the anterior region. Consistent with this suggestion, knockdown of cofilin expression in HeLa cells evoked various abnormalities associated with hyper activation of myosin II^32^, although these authors attributed this to direct competition for binding sites on actin protomers between cofilin and myosin II.

In summary, this study demonstrated a novel allosteric and cooperative mechanism for a mutually inhibitory relationship between myosin II and cofilin *in vitro*. In particular, allosteric negative regulation of cofilin activity by myosin II *in vivo* should be far more potent than the previously assumed direct competition mechanism, in which one molecule of bound motor domain can protect only one actin protomer from cofilin binding. Furthermore, ABPs that bind to the ends of actin filaments also affect the structure of the filament over a long distance^54^,^55^,^56^,^57^. Thus, ABP-induced allosteric and cooperative modification of the structure of actin filaments, and the resulting functional differentiation of the actin filaments, appear to be widespread.

## Methods

### Preparation of proteins

His-tagged human cofilin-mCherry was expressed in *E. coli* and purified using a Ni-affinity column, as detailed in the Supplementary Methods. Preparation of engineered chicken cofilin and labeling with fluorescein^26^ is also detailed in the Supplementary Methods. The following proteins were purified as described elsewhere: *Dd* actin^58^, *Dd* HMM-GFP^15^, *Dd* S1^59^, actin-S1 fusion protein^27^, cofilin-actin fusion protein^8^, and His-tagged *Dd* cofilin^60^. Rabbit sk actin and S1 were prepared by the method of Spudich and Watt^61^ and Margossion and Lowey^62^, respectively. *Dd*-actin was labeled with Cy3-succinimide and Alexa488-succinimide and sk actin with Alexa555-succinimide as described in the Supplementary Methods.

### Fluorescence microscopy

Flow cells coated with a positively charged lipid bilayer were prepared as described in the Supplementary Methods, and blocked with F-buffer 1 (25 mM imidazole, pH 6.5, 4 mM MgCl_2_, 25 mM KCl, 1 mM DTT, 10 mg/mL BSA). Each fluorescent or unlabeled *Dd* actin was polymerized for 6 h at 4 °C in F-buffer 1, diluted to 80 nM in F-buffer 1 containing 0.5 μM ATP, and introduced into the flow cell. After incubation for 5 min, excess actin filaments were washed away with F-buffer 1. F-buffer 1 containing 0.2 μM ATP and either or both 80 nM *Hs* cofilin-mCherry and 7 nM *Dd* HMM-GFP was then introduced, and incubated for 4 min. Finally, F-buffer 1 containing glucose, glucose oxidase and catalase was introduced and samples were observed under a fluorescence microscope (IX-70, Olympus, Tokyo, Japan) equipped with a Plan-Fluor 100×, 1.30 N.A. oil-immersion objective lens and a sCMOS camera (ORCA-Flash 2.8, Hamamatsu Photonics, Hamamatsu, Japan).

Procedures for TIRF observations are detailed in the Supplementary Methods. Briefly, filaments of Alexa555-labeled sk actin were first loosely immobilized on a positively-charged inner surface of flow cells. After blocking with 2 mg/mL BSA, 0.3 μM fluorescein-chicken cofilin in the observation buffer (40 mM KCl, 20 mM Pipes pH 6.8, 1 mM MgCl_2_, 0.5 mM EGTA, 10 mM DTT, 0.1 mM ATP, 2 mg/mL BSA, 10 mg/mL glucose, 100 μg/mL glucose oxidase and 20 μg/mL catalase) was gently introduced, and fluorescence images of Alexa555 and fluorescein were imaged simultaneously at a frame rate of 0.1 fps for 10 min. To examine the effects of S1 on binding of cofilin to actin filaments, observation buffer containing 0.3 μM or 1 μM S1 was first introduced into a flow cell with attached actin filaments. After incubation for 5 min, a mixture of 0.3 μM fluorescein-cofilin and either 0.3 μM or 1 μM S1 in the observation buffer was introduced, and time lapse images were taken as indicated above. For semi-quantitative comparison of the binding of fluorescein-cofilin, it was desirable to take snap-shot images of fluorescein without prior illumination with blue excitation light, in order to minimize complications caused by bleaching. Thus, snap-shot TIRF images of fluorescein and Alexa555 were taken at new fields after focusing using Alexa555 fluorescence. Image analyses, including background subtraction, were performed using ImageJ software.

### Cosedimentation assays

*Dd* actin (3 μM) or a mixture of 3 μM *Dd* actin and 1.5 μM actin-S1 fusion protein were polymerized in 70 mM KCl, 20 mM Pipes pH 6.5, 0.4 mM EGTA, 2 mM MgCl_2_, 2 mM ATP and 1 mM DTT at 22 °C for 1.5 h. *Dd* cofilin was then added at a final concentration of 3 μM, and incubated for 5 min. In the second experiment, 8 μM *Dd* actin or mixture of 8 μM actin and 10 μM cofilin-actin fusion protein were polymerized in 100 mM KCl, 2 mM Hepes pH 7.4, 0.5 mM EGTA, 2 mM MgCl_2_, 0.4 mM ATP, 0.1 mM DTT and 2 mg/mL BSA at 22 °C for 1.5 h. An equal volume of 3 μM *Dd* S1 in 2 mM ADP and 20 mM Pipes pH 6.5 was then added.

In each experiment, the mixtures were centrifuged at 250,000 × *g* for 10 min at 22 °C, and the supernatants and pellets were subjected to SDS-PAGE. In some experiments, BSA was included in buffers as a precautionary measure to prevent protein denaturation and absorption to the tube walls.

## Additional Information

**How to cite this article**: Ngo, K. X. *et al*. Allosteric regulation by cooperative conformational changes of actin filaments drives mutually exclusive binding with cofilin and myosin. *Sci. Rep.* **6**, 35449; doi: 10.1038/srep35449 (2016).

## Supplementary Material

Supplementary Information

Supplementary Video S1

Supplementary Video S2
