# Supplementary material for: Allosteric regulation by cooperative conformational changes of actin filaments drives mutually exclusive binding with cofilin and myosin
Source: Sci Rep. 2016 Oct 20;6:35449. doi: 10.1038/srep35449 (PMC5071871; doi:10.1038/srep35449)
Supplement: Supplementary Information [file srep35449-s1.pdf]

Supplementary information for:

Allosteric regulation by cooperative conformational changes of actin filaments drives mutually exclusive binding with cofilin and myosin

Kien Xuan Ngo*, Nobuhisa Umeki*, Saku T. Kijima*, Noriyuki Kodera, Hiroaki Ueno, Nozomi Furutani-Umezu, Jun Nakajima, Taro Q.P. Noguchi, Akira Nagasaki, Kiyotaka Tokuraku, and Taro Q.P. Uyeda

*: Contributed equally.

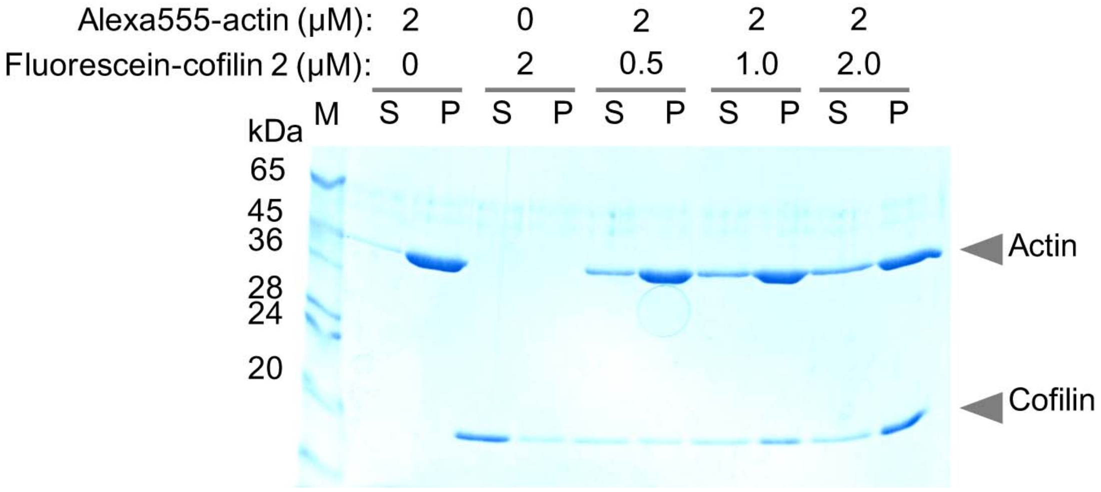

Supplementary Figure S1: Co-sedimentation of fluorescein-chicken cofilin 2 with Alexa555-actin filaments. A series of protein mixtures of Alexa555-actin filaments and fluorescein-cofilin 2 at different molar ratios were prepared, as shown above each lane. These mixtures were incubated for 5 min at 25 ^{o}C, followed by ultracentrifugation at 250,000 × *g* for 5 min at 25 ^{o}C. Supernatant (S) and pellet (P) fractions were collected, run separately on a 12% acrylamide SDS-PAGE gel, and subsequently stained with Coomassie Brilliant Blue.

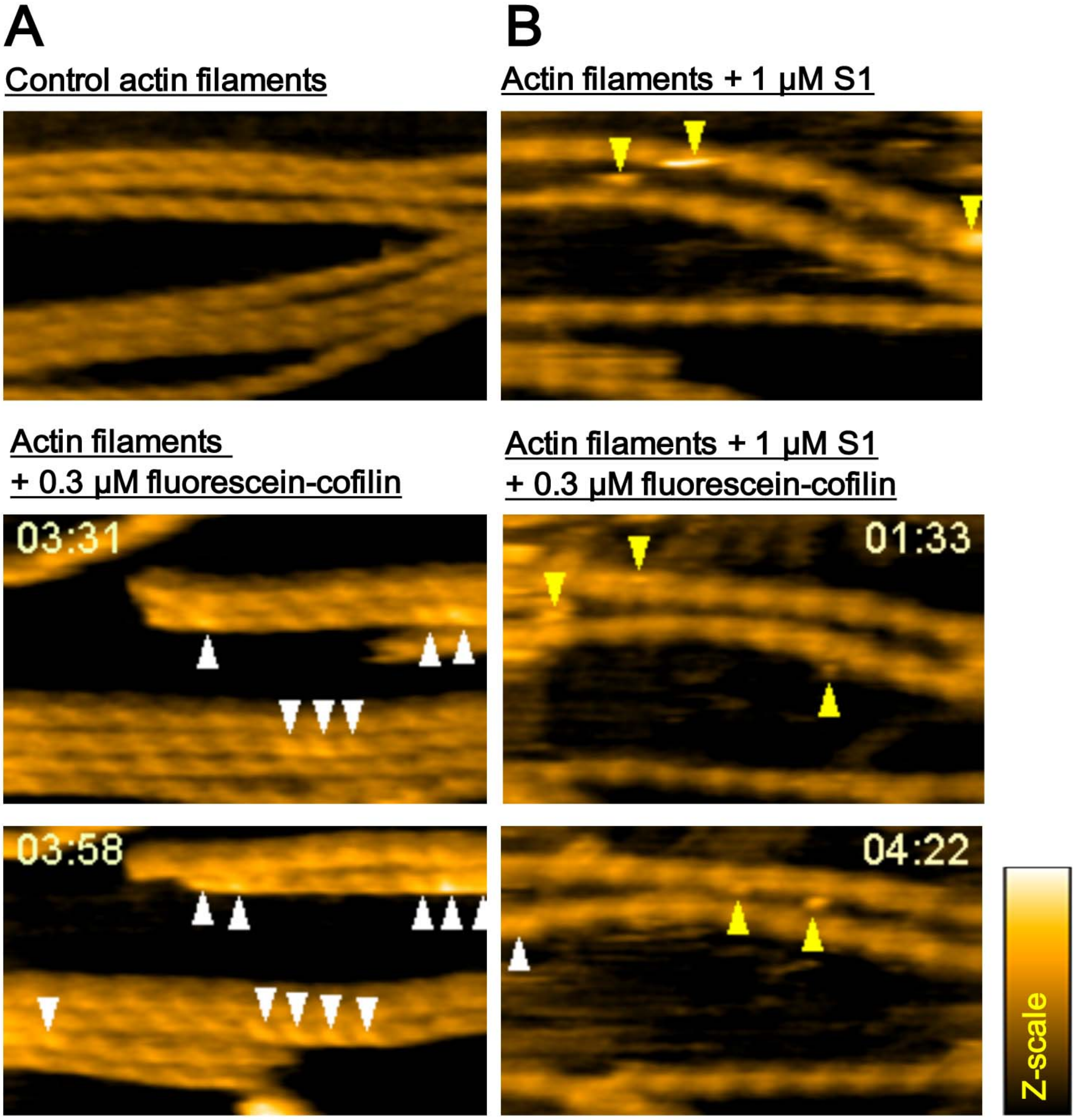

Supplementary Figure S2: Binding and cluster formation of cofilin along actin filament in the presence and absence of S1 as demonstrated by high-speed AFM. All experiments were acquired in F buffer containing 0.1 mM ATP, 40 mM KCl, 20 mM Pipes pH 6.8, 1 mM $MgCl_2$, 0.5 mM EGTA, 0.5 mM DTT. A: Still images of control actin filaments loosely immobilized on a positively charged lipid bilayer on mica and were observed by a laboratory-built high speed atomic force microscope [1] as described previously [2]. Observation was continued after the addition 0.3 μM fluorescein-cofilin 2. Time after the addition of cofilin is shown in min:sec. B: Similar to A, except 1 μM sk S1 was included in the buffer. White and yellow arrowheads show cofilin clusters and transiently binding individual S1 molecules, respectively. Note that, in the presence 0.1

mM ATP, actin filaments were only sparsely bound with S1, but formation of cofilin clusters was strongly inhibited. Imaging rates were 2 frames per second. Scanned sizes of all the images were 340 nm x 205 nm, and the Z-scale was 0-12 nm. Video sequence of A and B are shown in Supplementary Videos S1 and S2, respectively.

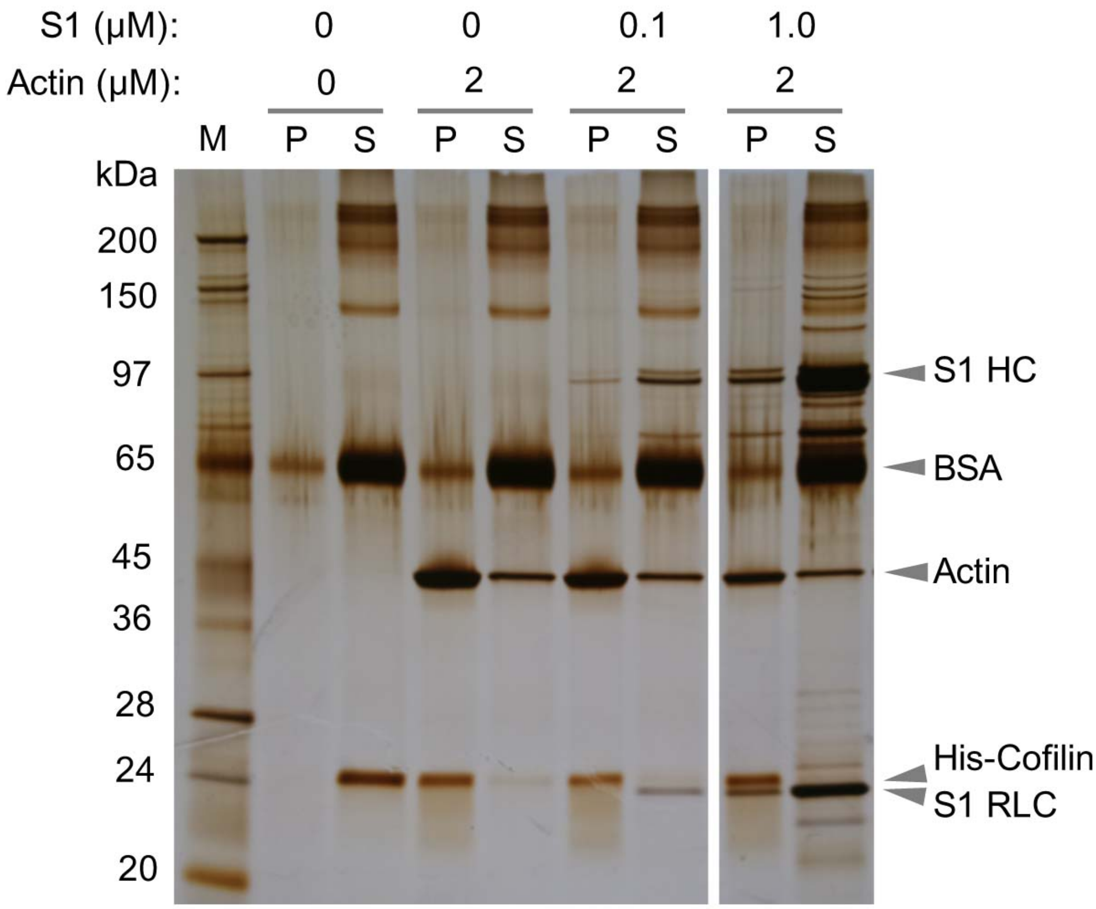

Supplementary Figure S3. Effects of S1 on cosedimentation of cofilin with actin filaments in the presence ATP and ADP. In this particular experiment, 2 μM sk actin filaments, 0.4 μM His-human cofilin, 0.1 or 1.0 μM sk muscle S1, 0.1 mg/mL BSA, 1 mM ATP and 1 mM ADP in 20 mM Pipes pH 6.8, 40 mM KCl, 4 mM $MgCl_2$, 0.5 mM EGTA, 0.5 mM DTT were incubated for 10 min at 22 ℃, and ultracentrifuged at 250,000 × *g* for 6 min at 22 ℃. The supernatant and pellet fractions were separately run on a 5~20% gradient gel, and silver-stained. Inclusion of BSA as carrier was essential to prevent absorption of 0.4 μM His-cofilin to the wall of centrifuge tubes, but this resulted in a number of anomalous extra bands even though we used purified BSA (Sigma A4378). Trace contaminants present in our S1 preparation were also evident in the supernatant fraction containing 1 μM S1. Nonetheless, it is obvious that 0.1 or 1 μM S1 did not inhibit cosedimentation of His-cofilin with sk actin filaments. We tested various other conditions, including omitting ADP, changing the concentration of actin or cofilin, use of cofilin without the His-tag, longer or shorter incubation before

ultracentrifugation, but were unable to find a condition under which S1 significantly inhibits cosedimentation of cofilin with sk actin filaments in the presence of ATP.

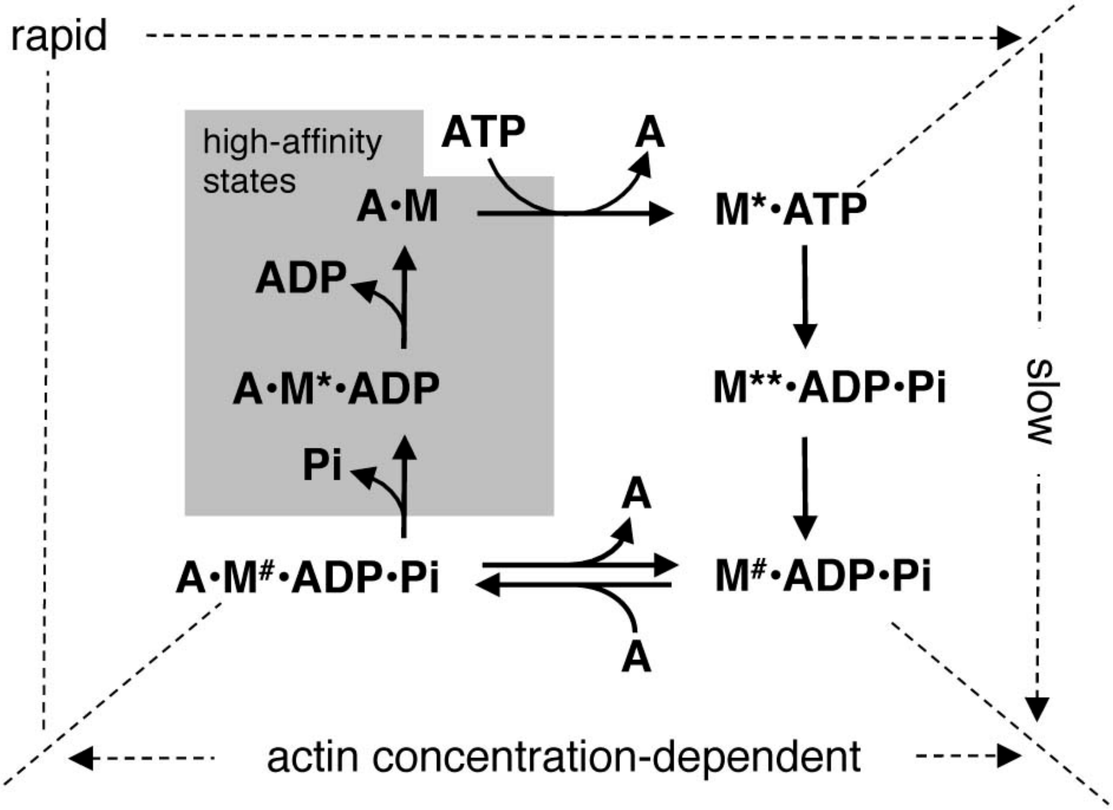

Supplementary Figure S4. Scheme of actin-activated ATPase cycle of S1, adopted and simplified from Stein *et al.* [3,4]. A and M represent actin protomer and S1, respectively, and M*, M** and $M^{\#}$ represent different conformations of S1. ATP binding rapidly dissociates A•M rigor(-like) complex. S1 spends relatively long time in the low affinity states for actin during ATP hydrolysis from M*•ATP to M**•ADP•Pi and subsequent isomerization from M*•ADP•Pi to $M^{\#}$•ADP•Pi. In the presence of saturating concentration of actin, the isomerized $M^{\#}$•ADP•Pi rapidly binds to actin filaments, triggering Pi and ADP release accompanied by swinging motion of the lever arm. The A•M*•ADP and A•M states have strong binding affinities for actin (gray box). Therefore, $M^{\#}$•ADP•Pi can be regarded as "non-refractory" [3] or the state that is capable of forming strong binding with actin. When the concentration of actin is lower, the semi-stable $M^{\#}$•ADP•Pi state persists until either it encounters actin filaments or until Pi is spontaneously released, which is very slow. Thus, the fraction of S1 molecules that is in the state capable of forming strong binding with actin is higher when the actin concentration is lower.

This means that the frequency of actin protomer being transiently but strongly bound with S1 in the presence of ATP should be a function of not only the concentration of S1 but also the concentration of actin, i.e., the molar ratio of S1 to actin in the reaction mixture.

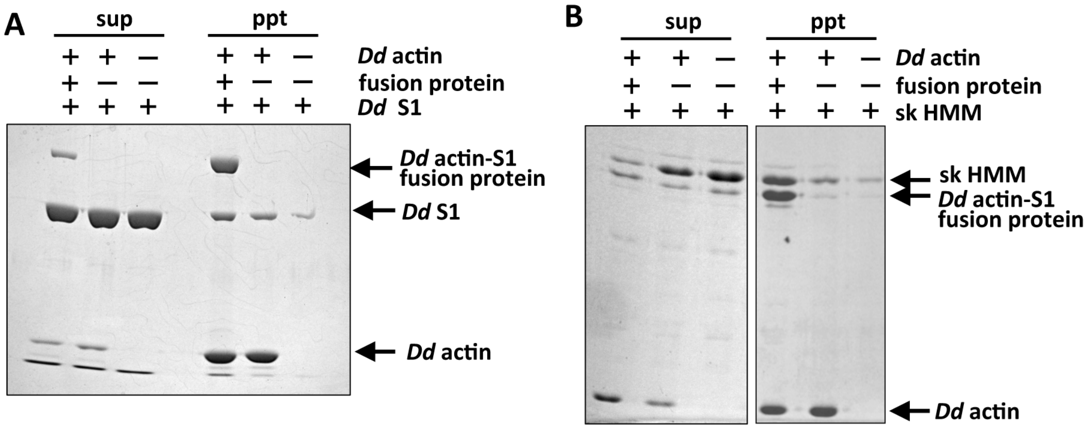

Supplementary Figure S5. Cosedimentation of motor domains of myosin II with copolymers of actin and actin-S1 fusion protein. (A) Cosedimentation of *Dd* S1 with copolymers of *Dd* actin and actin-S1 fusion protein. *Dd* S1 at 1.5 µM was mixed with either 4 µM *Dd* actin homopolymer or copolymer (4 µM *Dd* actin and 1.5 µM actin-S1 fusion protein) in 70 mM KCl, 2 mM $MgCl_2$, 0.4 mM EGTA, 2 mM Hepes pH 7.4, 0.2 mM DTT and 0.2 mM ATP. Immediately after the addition of 1 mM ADP and 10 mM MgATP, the mixtures were centrifuged at 250,000 × *g* for 10 min at 22 °C. (B) Rabbit sk HMM at 0.6 µM was mixed with either 3 µM *Dd* actin homopolymer or copolymer (3 µM *Dd* actin and 1.5 µM actin-S1) in 70 mM KCl, 2 mM $MgCl_2$, 0.4 mM EGTA, 2 mM Hepes pH 7.4, 0.2 mM DTT, 1 mM ADP and 0.2 mM ATP. The mixtures were immediately centrifuged at 250,000 × *g* for 10 min at 22 °C. In each experiment, the resultant supernatants and pellets were run on SDS-PAGE. In both cases, a larger amount of *Dd* S1 or sk HMM cosedimented with the copolymers than with the actin homopolymers, suggesting that the motor domains of myosin II have higher affinities for the copolymers. However, we were unable to rule out the possibility that this was in part due to consumption of ATP by the actin-S1 fusion protein. Nonetheless, it is evident that actin subunits in copolymers have the potential to bind the motor domains of myosin II.

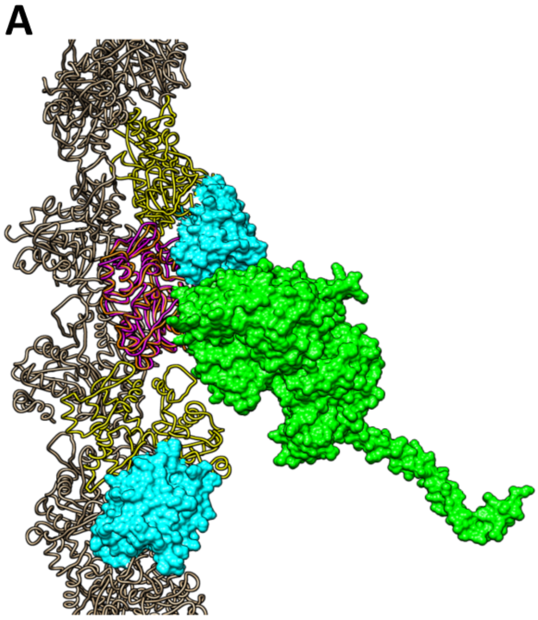

Supplementary Figure S6. Modeling of rigor S1 binding onto cofilin-decorated actin filaments. From the structure of sk actin filaments fully bound with human cofilin 2 (PDB ID:3J0S [5]), all cofilin molecules were removed except for two (cyan) interrupted by one unoccupied binding site (orange). To this orange actin subunit, an sk actin subunit (magenta) complexed with chicken sk S1 (green; only the heavy chain is shown) taken from the modeled structure of S1-bound actin filaments (PDB ID: 1O1F, [6]) was superimposed using the MatchMaker function of Chimera software. (A): side view, (B): oblique view. In this model, there is a large space between S1 and the lower cofilin molecule, and S1 is just touching the upper cofilin molecule (arrow in B). This model needs to be interpreted with some precaution, however, because the atomic structures of actin in the two original models differ significantly, and the superimposition is somewhat ambiguous. In addition, the proteins used in our cosedimentation experiments were derived from *Dd*. *Dd* cofilin is 29 amino acid residues smaller than human cofilin 2 used in the modeling [5], and the motor domain of *Dd* myosin II is 19 residues smaller than that of chicken sk myosin used in the modeling [6], both excluding the purification tags. Thus, the difference in protein sources is unlikely to overestimate the space between the two proteins.

Supplementary Video S1: Binding and cluster formation of fluorescein-cofilin 2 along actin filaments in the absence of S1 as demonstrated by high-speed AFM. Actin filaments were first imaged in a buffer that contained 0.1 mM ATP. Subsequently, 0.3 μM fluorescein-cofilin 2 was added at time zero, and imaging was continued. White arrowheads show clusters of cofilin. Imaging rate was 2 frames per second and the movie is played at 5 frames per second. For more details, refer to the legend to Supplementary Figure S2A.

Supplementary Video S2: Inhibition of cluster formation of cofilin along actin filaments by transiently binding S1 in the presence of ATP, as demonstrated by high-speed AFM. Actin filaments were first imaged in a buffer that contained 0.1 mM ATP. Subsequently, 1 μM S1 was added. This was followed by the addition of 0.3 μM fluorescein-cofilin 2 at time zero, and imaging was continued. White and yellow arrowheads show cofilin clusters and transiently binding S1 molecules, respectively. Imaging rate was 2 frames per second and movie is played at 5 frames per second. For more details, refer to the legend to Supplementary Figure S2B.

Supplementary Table S1. Inhibition of cofilin binding to actin filaments by S1 in the presence of ATP.

Concentration of S1 (μM)	Normalized ratio (green/red), mean ± SD	Total length of measured filaments (μm)	Number of measured filament segments
0	100 ± 24	228	37
0.3	71 ± 25	280	32
1.0	33 ± 18	227	34

For experimental details, refer to Fig. 3 in the main text. Ratios of green/red fluorescence intensities were measured at each pixel along straight segments of randomly selected filaments, as shown in Fig. 3D-F, and mean values were computed for each segment. Mean and SD of the mean ratio values of 32-37 filament segments were then computed for each of the three experimental conditions. Finally, the mean and SD of each condition are normalized against those in the absence of S1. Each experimental condition contained three independent preparations.

Supplementary Methods

Preparation of fluorescent cofilin

Human cofilin fused with mCherry at the C-terminus was prepared as follows. cDNAs encoding human cofilin 1 and mCherry [7] were subcloned at the *Bam*HI-*Eco*RI and the *Eco*RI-*Xba*I sites of pColdI (Takara Bio, Otsu, Japan), respectively. The resultant plasmid was used to transform Rosetta (DE3) *E. coli* cells (Novagen). Expression was induced as indicated by the manufacturer's instructions. The expressed protein was purified using Ni-NTA resin (Qiagen), dialyzed against 10 mM Hepes pH 7.5, 100 mM NaCl, and snap-frozen in liquid nitrogen.

Chicken cofilin chemically labeled with fluorescein was prepared as follows. Chicken cofilin 2 with two extra Cys residues at the N-terminus was made as described previously [8], except that the coding sequence was placed in a pColdI vector engineered to have a TEV cleavage site between the His-tag and the multi-cloning site. After expression in *E. coli*, purification using Ni-NTA resin, cleavage with the His-tagged TEV protease and dialysis against 10 mM Hepes, 10 mM imidazole, pH 7.4, 50 mM NaCl, 0.1 mM EDTA, 0.7 mM 2–mercaptoethanol, and 0.05% NaN_3, the recombinant chicken cofilin was separated from the cleaved His-tag-TEV recognition sequence and the His-TEV protease by passing through a small volume of Ni-NTA resin. Purified cofilin was dialyzed against a buffer containing 10 mM Hepes pH 7.4, 50 mM KCl, 0.1 mM Tris(2-carboxyethyl)phosphine hydrochloride (TCEP) and 0.05% NaN_3. Cofilin was diluted to 2 mg/mL in the same buffer, and 20 mM iodoacetamide fluorescein prepared in DMSO was gently added until the final molar ratio was 0.8 to 1. The labeling reaction was carried out on ice in the dark for 0.5 h, and was stopped by adding 10 mM DTT. Free dye was removed by dialysis against the same buffer but containing 0.1 mM DTT instead of TCEP. After clarification by centrifugation at 286,000 × *g* at 4 °C for 5 min, aliquots of fluorescein-labeled cofilin were snap-frozen in liquid N_2 and stored at -80 °C. The labeling efficiency was ~80%.

Fluorescence labeling of actin

Dd actin was polymerized in 10 mM Hepes pH 7.4, 100 mM KCl, 2 mM $MgCl_2$, 0.2 mM ATP, and 0.5 mM DTT and diluted to 20 μM with this buffer containing a final concentration of 120 μM Cy3-succinimidyl ester (Lumiprobe, Hallanadale Beach, FL, USA) or 160 μM Alexa Fluor 488 carboxylic acid succinimidyl ester (Thermo

Fisher, Yokohama, Japan) in 10 mM Pipes pH 6.8, 100 mM KCl, 2 mM $MgCl_2$, 0.2 mM ATP, and 0.5 mM DTT. After incubation for 12 h on ice, unreacted fluorophore was removed by ultracentrifugation at 286,000 × *g* for 10 min. The resultant pellet was dissolved in and dialyzed against G-buffer (2 mM Hepes pH 7.4, 0.2 mM $CaCl_2$, 0.2 mM ATP, 1 mM DTT) for 2 days at 4 °C. The supernatant after clarification by ultracentrifugation at 286,000 × *g* for 10 min was snap-frozen in small aliquots and stored in liquid nitrogen. The labeling efficiency was 54% for Cy3-actin and 47% for Alexa488-actin.

Skeletal actin was labeled with Alexa Fluor 555 carboxylic acid succinimidyl ester (Thermo Fisher). Briefly, actin was dialyzed against G buffer (2 mM Hepes pH 8.3, 0.2 mM $CaCl_2$, 0.2 mM ATP) at 4 °C. Actin was diluted to 48 µM with G buffer, and 20 mM Alexa555 in DMSO was gently added to a final molar ratio of 2:1. The labeling reaction was carried out on ice for 16 h and was stopped by adding 20 mM Tris-Cl, pH 7.4. Labeled actin was polymerized by the addition of 2 mM $MgCl_2$ and 100 mM KCl for 2 h at room temperature, and was collected by centrifugation at 286,000 × *g* for 1 h at 4 °C. The labeled actin pellet was washed twice by F buffer (10 mM Tris-Cl, pH 7.4, 100 mM KCl, 2 mM $MgCl_2$, 1 mM DTT, 0.5 mM ATP), and was then dissolved in G buffer and dialyzed against G buffer for 24 h at 4 °C. After clarification by centrifugation at 286,000 × *g* for 5 min at 4 °C, aliquots of labeled actin were snap-frozen and stored in liquid N_2. The labeling efficiency was ~85%.

Preparation of positively charged glass surfaces

Small unilamellar vesicles (SUVs) and a glass-supported lipid bilayer were prepared as described previously [9,10]. The lipid composition was 1,2-dipamitoyl-*sn*-glycero-3-phosphocholine (DPPC; Avanti Polar Lipids, Alabaster, AL) and 1,2-dipalmitoyl-3-trimethylammonium-propane (DPTAP; Avanti Polar Lipids) at a weight ratio of 9:1. SUVs were dispersed in Milli-Q water at 2 mg/mL and stocked at -20°C. Before use, SUVs were diluted in 5 mM $MgCl_2$ to 0.5 mg/mL and sonicated with a bath sonicator for 1 min. An aliquot of the sonicated SUVs was introduced into a flow cell made of a clean coverslip and a glass slide separated by double-sided tape, and incubated for 1 h at 36 °C in a humid environment. The inside of flow cells was rinsed with buffer to remove excess SUVs and lipid bilayers before use.

For TIRFM experiments, glass slides and cover slips were first cleaned by

sonication in detergent for 30 min, followed by cleaning in 3 M KOH solution for 3 h, extensive washing in milli-Q water in a bath sonicator, and drying at 50 °C. Glass slides were silanized by dipping in 2 mg/mL (3-aminopropyl)triethoxysilane diluted in water for 5 min, washed gently by milli-Q water three times, and dried at 50 °C for 1 h, prior to making a flow chamber using double-sided tape.

TIRF microscopy

An Olympus total internal reflection (TIRF) system was used with an inverted microscope (IX71, Olympus) and an objective lens (Apo 100x OHR; NA 1.65, Olympus). To observe proteins labeled with fluorescein or Alexa555, we used a 488-nm (COHERENT, Tokyo, Japan) and 543-nm (Showa Optronics, Tokyo, Japan) solid state laser, respectively. Our TIRFM system was equipped with an Orca-Flash 4.0 CMOS camera (Hamamatsu) and shutters (Ludl Electronics, Hawthorne, NY, USA) to control each light source by HC image software (Hamamatsu).

Frozen stocks of Alexa555-labeled sk actin, fluorescein-labeled chicken cofilin, and sk S1 were first thawed and clarified by centrifugation at 286,000 × *g* for 5 min at 4 °C. Labeled actin was polymerized at 0.5 mg/mL in buffer A that contained 40 mM KCl, 20 mM Pipes pH 6.8, 1 mM $MgCl_2$, 0.5 mM EGTA, 1 mM DTT, and 0.1 mM ATP at room temperature for at least 1 h. Actin filaments were diluted to 40 nM in buffer A and were introduced into a flow cell made of a glass slide and a coverslip whose surface had been modified with amino-silane. After incubation for 2 min, flow cells were washed twice by buffer A to remove unbound actin filaments, followed by buffer B (buffer A containing 2 mg/mL BSA and 10 mM DTT) for 2 min, and were finally filled with observation buffer (buffer B containing 10 mg/mL glucose, 100 μg/mL glucose oxidase and 20 μg/mL catalase). In the control experiment, 300 nM fluorescein-cofilin prepared in the observation buffer was gently poured into a flow cell with attached actin filaments, and fluorescence images of Alexa555 and fluorescein were captured simultaneously at a frame rate of 0.1 fps for 10 min. To examine the effects of S1 on binding of cofilin to actin filament, observation buffer containing 300 nM or 1 μM S1 was first introduced into a flow cell with attached actin filaments. After incubation for 5 min, a mixture of 300 nM fluorescein-cofilin and either 300 nM or 1 μM S1 in the observation buffer was introduced. To follow the kinetics of cofilin binding, fluorescence images of Alexa555 and fluorescein were captured

simultaneously at a frame rate of 0.1 fps for 10 min. For semi-quantitative comparison of the binding of fluorescein-cofilin, it was desirable to take snap-shot images of fluorescein without prior illumination with blue laser light, in order to minimize complications caused by bleaching. Thus, in some experiments, snap-shot TIRF images of fluorescein and Alexa555 were taken at new fields after focusing using Alexa555 fluorescence.

Supplementary References

1 Ando, T., Uchihashi, T. & Kodera, N. High-speed AFM and applications to biomolecular systems. *Annu Rev Biophys* **42**, 393-414, doi:10.1146/annurev-biophys-083012-130324 (2013).

2 Ngo, K. X., Kodera, N., Katayama, E., Ando, T. & Uyeda, T. Q. P. Cofilin-induced unidirectional cooperative conformational changes in actin filaments revealed by high-speed atomic force microscopy. *Elife* **4**:e04806, doi:10.7554/eLife.04806 (2015).

3 Stein, L. A., Schwarz, R. P., Jr., Chock, P. B. & Eisenberg, E. Mechanism of actomyosin adenosine triphosphatase. Evidence that adenosine 5'-triphosphate hydrolysis can occur without dissociation of the actomyosin complex. *Biochemistry* **18**, 3895-3909 (1979).

4 Stein, L. A., Chock, P. B. & Eisenberg, E. Mechanism of the actomyosin ATPase: effect of actin on the ATP hydrolysis step. *Proc Natl Acad Sci U S A* **78**, 1346-1350 (1981).

5 Galkin, V. E. *et al.* Remodeling of actin filaments by ADF/cofilin proteins. *Proc. Natl. Acad. Sci. USA.* **108**, 20568-20572, doi:10.1073/pnas.1110109108 (2011).

6 Chen, L. F., Winkler, H., Reedy, M. K., Reedy, M. C. & Taylor, K. A. Molecular modeling of averaged rigor crossbridges from tomograms of insect flight muscle. *J Struct Biol* **138**, 92-104, doi:10.1016/S1047-8477(02)00013-8 (2002).

7 Shu, X., Shaner, N. C., Yarbrough, C. A., Tsien, R. Y. & Remington, S. J. Novel chromophores and buried charges control color in mFruits. *Biochemistry* **45**, 9639-9647, doi:10.1021/bi060773l (2006).

8 Nagaoka, R., Kusano, K., Abe, H. & Obinata, T. Effects of cofilin on actin filamentous structures in cultured muscle cells. Intracellular regulation of cofilin action. *J Cell Sci* **108 (Pt 2)**, 581-593 (1995).

9 Uchihashi, T., Kodera, N. & Ando, T. Guide to video recording of structure dynamics and dynamic processes of proteins by high-speed atomic force microscopy. *Nat Protoc* **7**, 1193-1206, doi:10.1038/nprot.2012.047 (2012).

10 Yamamoto, D. *et al.* High-speed atomic force microscopy techniques for observing dynamic biomolecular processes. *Methods Enzymol* **475**, 541-564, doi:10.1016/S0076-6879(10)75020-5 (2010).
